# Successful Management of Ludwig's Angina due to Dental Implant Displacement: A Rare Case Report

**DOI:** 10.1155/2020/6934286

**Published:** 2020-02-19

**Authors:** Lincoln Lara Cardoso, Giovanni Gasperini, Leandro Carvalho Cardoso, Guilherme Romano Scartezini, Annika Ingrid Maria Soderberg Campos, Heloisa Fonseca Marão

**Affiliations:** ^1^Department of Oral and Maxillofacial Surgery, Aparecida de Goiânia Hospital, Goiânia, Goiás, Brazil; ^2^Department of Implantology, University of Santo Amaro, São Paulo, São Paulo, Brazil

## Abstract

Dental implant surgery is a common procedure in oral and maxillofacial surgery practices. Extensive training, skill, and experience allow this procedure to be performed with an atraumatic approach, but like any surgical technique, it is subject to accidents and complications. This is an unusual clinical case of an accidental displacement of an implant into the submandibular space that progressed to Ludwig's angina, and it has not yet been described in the literature. This case report describes a clinical case of dental implant displaced into the submandibular space after healing cap removal. After seven days, it progressed to Ludwig's angina. The removal was performed through extraoral access in the submandibular area by using hemostatic forceps and radioscopic technique. After implant removal, the clinical case showed a satisfactory repair emphasizing the importance of a meticulous clinical planning to achieve an appropriate treatment plan, which is essential for a favorable prognosis. Therefore, prevention and management of displaced objects requires proper planning and surgical technique.

## 1. Introduction

Implant-rehabilitation protocols are considered a treatment with good surgical and prosthetic predictability with high success rates [1]. Although dental implant surgery is a simple, predictable, and safe procedure, accidents and complications may occur [2]. The most frequent complications of titanium dental implant treatment are infection, implant rejection, and implant displacement. Displacement of implants into the maxillary sinus is a common complication encountered in oral and maxillofacial surgery, but the implant can also shift into the facial spaces, especially the infratemporal, buccal, sublingual, and submandibular fossae [3–5].

Ludwig's angina was originally described by the German army physician Wilhelm Frederick von Ludwig in 1836. This is a type of soft tissue infection (cellulitis) involving three compartments on the floor of the mouth [6]. The treatment of Ludwig's angina should consist of airway maintenance, adequate antibiotic therapy, and intraoral or extraoral surgical drainage when necessary [7]. Although displacement of objects may occur in the practice of almost all procedures performed in the oral and maxillofacial surgery, there is no report in the literature of dental implant displacement that had led to Ludwig's angina. Thus, the aim of this article is to present a rare case of Ludwig's angina due to displacement of dental implant into the submandibular space. Therefore, early treatment and correct management are recommended because this is a clinical diagnosis with unpredictable progression.

## 2. Case Report

A 47-year-old man underwent oral surgery in a private dental clinic for dental implant in the posterior region of the mandible. According to the surgeon's and patient's history, the implant (Titamax implants 3.75 × 11 mm, Neodent, Curitiba, Paraná, Brazil) was placed at the region of the first lower right molar, but fenestration of the lingual cortical plate required simultaneous bone regeneration by using lyophilized bovine bone grafts (Genox Inorganic, Baumer, SP, Brazil) and collagen membrane (GenDerm, Baumer, SP, Brazil). The implant primary stability was checked with a torque wrench used at a force of 32 N·cm, and the healing cap was placed. There was no postoperative complication during the period of bone repair.

After 120 days, during the healing cap removal, the implant was accidentally displaced into the submandibular space. Although the lingual access was performed through intrasulcular flap in the same session, the implant was not localized. Therefore, the intraoral access was closed and the patient medicated with amoxicillin (500 mg, 08/08 h/07days), nimesulide (100 mg, 12/12 h/03days), and dipyrone sodium (500 mg, 06/06 h). The patient was instructed to undergo a computed tomography (CT) scan of the mandible for reassessment and removal of the implant.

**Figure 1:**
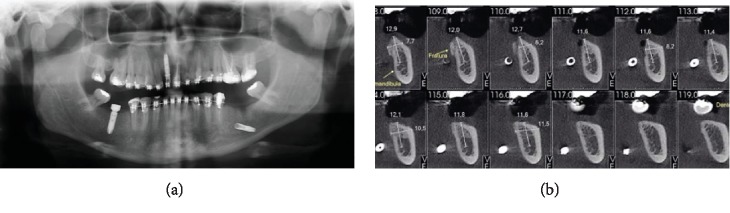
(a) Panoramic radiographic image. (b) Cone beam computed tomographs in sagittal sections showing displacement of the dental implant and fracture of the mandibular lingual cortical bone adjacent to the region of tooth #36.

**Figure 2:**
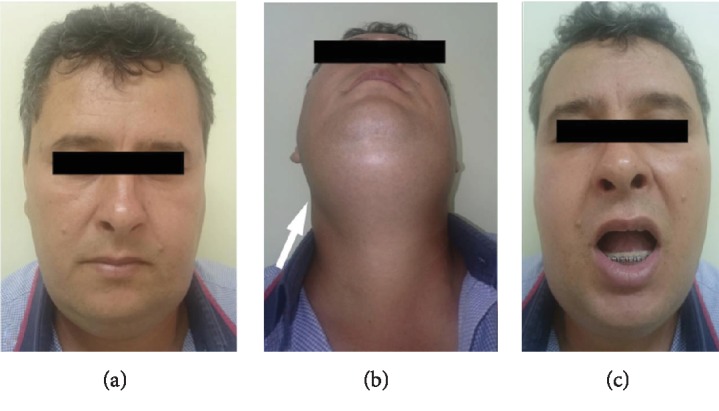
(a) Clinical aspect of the Ludwig's angina. (b) Arrow shows anterior neck edema. (c) Reduced mouth opening.

Panoramic radiographic and CT scan examinations showed displacement of the dental implant into the submandibular space and fracture of the mandibular lingual cortical bone adjacent to the region of tooth #36 (Figures 1(a) and 1(b)). The patient was advised to return within 48 hours, but he did it only after seven days complaining of pain, swelling in the face, fever, and difficulty swallowing. After an initial evaluation, the patient was referred urgently to the Buccomaxillofacial Surgery Service of the Aparecida de Goiânia Hospital, Goiânia, Goiás, Brazil (Figures 2(a)–2(c)).

On physical examination, the patient presented consistent swelling in the submental region. In the submandibular and sublingual spaces, there was presence of painful symptomatology on local palpation bilaterally, trismus, mouth opening of approximately 20 mm, dysphonia, pain on cervical palpation, and intraoral purulent drainage, also affecting the floor of the mouth. In view of the clinical symptoms, laboratory tests and hospitalization were requested. Laboratory tests confirmed the infection, and Ludwig's angina was diagnosed.

**Figure 3:**
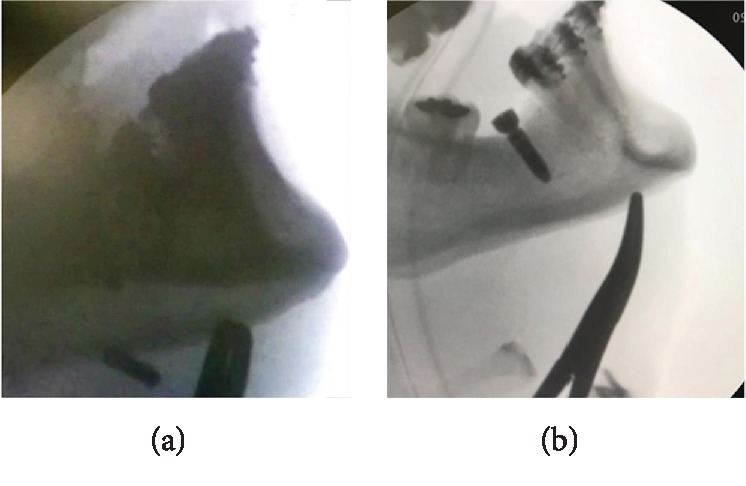
(a) X-ray profile showing location of the dental implant in the submandibular region. (b) X-ray showing clamping and removal of the dental implant.

After lingual flap retraction at the region of implant placement, detachment of the mucoperiosteum was performed for exploration of the area, but the dental implant was not found. Due to the failure of the intraoperative procedure, it was decided to use the surgical arch for radiographic shots in profile. At this time, it was verified that the implant had shifted to the submandibular space (Figure 3(a)). Following the treatment, extraoral access was performed in the submandibular area by using hemostatic forceps and radioscopic technique, thus allowing the dental implant to be clamped and removed (Figure 3(b)).

**Figure 4:**
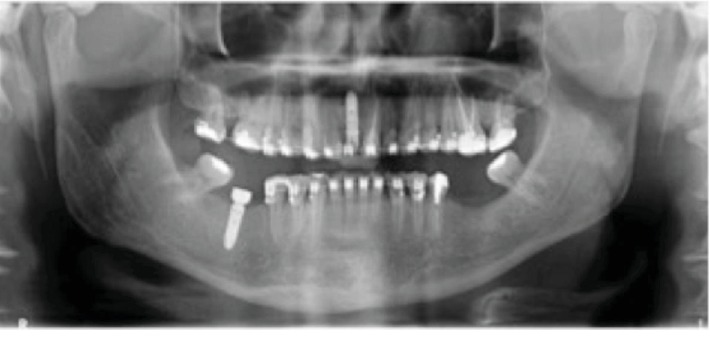
Postoperative panoramic radiograph without other complaints after 7 days.

After removal, a Penrose drain was inserted into the bilateral submandibular region and the patient remained hospitalized for 72-hour follow-up, receiving ceftriaxone (1 g, 12/12 h), clindamycin (600 mg, 06/06 h), dexamethasone (5 mg, 12/12 h), tenoxicam (20 mg, 12/12 h), and sodium dipyrone (2 cc, 06/06 h). The Penrose drain was removed after 48 hours. The patient's postoperative recovery was uneventful, with regression of signs and symptoms, and he was discharged from the hospital with clindamycin (600 mg, 06/06 h for 7 days) prescription. After 7 days, the patient returned for reassessment. Clinical and radiographic examinations were performed, and the patient presented neither signs of infection nor limitation of mouth opening and pain complaints (Figure 4).

## 3. Discussion

Reports of accidental implant displacement often indicate that this is located in the upper craniofacial structures such as the maxillary sinus, the ethmoid sinus, or the orbital floor [8]. However, an implant displaced into the lower spaces with evolution for Ludwig's angina has not been reported in the literature.

Quality of bone tissue found in the posterior regions of the maxilla and mandible, anatomical variations, inadequate surgical technique, inexperienced surgeon, insufficient planning, bone resorption, improper occlusal forces, and bone deficiencies could all cause implant displacement complications [3, 9].

The submandibular fossa represents a high risk zone during placement of dental implants due to the possibility of fenestration or perforation of the lingual cortical plate. When the submandibular fossa is pronounced, the implants should be angled correctly for proper perforation [10, 11]. According to Kim et al. (2015), a dental implant can be dislodged between the alveolar bone and lingual flap when the mandibular lingual cortical bone is absent, resulting in the risk of it sinking into the lower soft tissue [12].

Thus, CT scan allows characterization of the submandibular fossa anatomy and provides important information to evaluate the posterior mandible region for implant placement [13]. Height and width of the bone, mandibular canal location, and anatomical characteristics of the submandibular fossa will define the implant length and diameter, as well as the preparation angle for implant [14]. In the present case report, the CT scan was not performed to plan the implant preparation, being only used after dental implant displacement into the submandibular space for location and surgical removal of it.

The infection evolution to Ludwig's angina is possibly related to factors such as lingual cortical plate fracture at the region of tooth #36, inadequate use of antibiotic therapy, no return within 48 hours, and patient's poor oral hygiene and insufficient rest.

Complications due to displacement of dental implants into the submandibular region can be avoided with a correct surgical planning through CT scan. Therefore, the placement of implants of inadequate length and diameter and in the wrong three-dimensional position should be avoided. In the present case report, we believe that the main cause for implant displacement was the surgeon's inexperience, resulting in a wrong surgical planning. The implant was placed very lingually (absence of CT), which caused fenestration and fracture of the lingual cortical plate. Consequently, there was no primary stability during the osseointegration period and the implant invaded the submandibular space when the healing cap procedure was performed.

## 4. Conclusion

In conclusion, the displacement of dental implants into the submandibular space evolving into Ludwig's angina is a rare complication in implant dentistry. Early intervention to maintain airways preserved, including drainage and removal of the dental implant, is mandatory in the treatment. In this case report, the radioscopic equipment has proved to be efficient for removal of the dental implant.
